# Vulnerability to Cyberattacks and Sociotechnical Solutions for Health Care Systems: Systematic Review

**DOI:** 10.2196/46904

**Published:** 2024-05-31

**Authors:** Pius Ewoh, Tero Vartiainen

**Affiliations:** 1 School of Technology and Innovations Information Systems Science University of Vaasa Vaasa Finland

**Keywords:** health care systems, cybersecurity, sociotechnical, medical device, secure systems development, training, ransomware, data breaches, protected health information, patient safety

## Abstract

**Background:**

Health care organizations worldwide are faced with an increasing number of cyberattacks and threats to their critical infrastructure. These cyberattacks cause significant data breaches in digital health information systems, which threaten patient safety and privacy.

**Objective:**

From a sociotechnical perspective, this paper explores why digital health care systems are vulnerable to cyberattacks and provides sociotechnical solutions through a systematic literature review (SLR).

**Methods:**

An SLR using the PRISMA (Preferred Reporting Items for Systematic Reviews and Meta-Analyses) was conducted by searching 6 databases (PubMed, Web of Science, ScienceDirect, Scopus, Institute of Electrical and Electronics Engineers, and Springer) and a journal (*Management Information Systems Quarterly*) for articles published between 2012 and 2022 and indexed using the following keywords: “(cybersecurity OR cybercrime OR ransomware) AND (healthcare) OR (cybersecurity in healthcare).” Reports, review articles, and industry white papers that focused on cybersecurity and health care challenges and solutions were included. Only articles published in English were selected for the review.

**Results:**

In total, 5 themes were identified: human error, lack of investment, complex network-connected end-point devices, old legacy systems, and technology advancement (digitalization). We also found that knowledge applications for solving vulnerabilities in health care systems between 2012 to 2022 were inconsistent.

**Conclusions:**

This SLR provides a clear understanding of why health care systems are vulnerable to cyberattacks and proposes interventions from a new sociotechnical perspective. These solutions can serve as a guide for health care organizations in their efforts to prevent breaches and address vulnerabilities. To bridge the gap, we recommend that health care organizations, in partnership with educational institutions, develop and implement a cybersecurity curriculum for health care and intelligence information sharing through collaborations; training; awareness campaigns; and knowledge application areas such as secure design processes, phase-out of legacy systems, and improved investment. Additional studies are needed to create a sociotechnical framework that will support cybersecurity in health care systems and connect technology, people, and processes in an integrated manner.

## Introduction

### Background

Cybersecurity in health care systems entails the safeguarding of electronic information and assets against unauthorized access, use, and disclosure [1]. The main objective of cybersecurity in health care systems is to protect the privacy, integrity, and accessibility of health information to provide secure health care services. Despite the digital transformation in health care delivery, health care organizations are facing increasing challenges and crises, which include data breaches of patient health information and vulnerability in their critical infrastructure [2]. Research has highlighted that health care systems are becoming more vulnerable to cyberattacks as technology advances [3]. Furthermore, the internet and its diverse nature and connection to the delivery of telehealth and continuous health care services create multiple points of access for cyberattacks [4,5].

In high-income countries such as Finland, the United States, and the United Kingdom, integrated technology is used to monitor and manage health care systems. For instance, at least 10 to 15 medical devices are linked to each patient’s electronic bed in a public hospital [6]. These complexities increase the susceptibility of health care networks to cyberattacks [6,7]. Studies conducted through the simulation of medical devices have similarly revealed that pacemakers and pulse oximeters can be hacked and compromised without a physician’s knowledge [8,9]. Ransomware is another type of man-made malware that can disrupt health care systems by infecting computer systems, locking people out of their files, and then demanding a ransom payment in exchange for access to those files [10,11]. Cyberattackers can publish the exposed health information to the web or sell it on the dark web [12]. This type of attack can result in breaches of patient privacy, subjecting health care organizations to fines that are consistent with human health service regulations and European General Data Protection Regulation (GDPR) policies for data breaches. For example, research has shown that, between 2012 and 2022, more than US $128,244,290 million in fines were paid in the United States alone for violations of Health Insurance Portability and Accountability Act laws on data breaches against health care organizations [13]. Although these fines were derived from no less than 111 health care organizations, many organizations have failed to report breaches.

Cybersecurity education is seriously lacking [14,15]. Moreover, a critical problem with cybersecurity in health care systems is the lack of involvement or recruitment of people with expertise and training in cybersecurity [16], resulting in considerable neglect of the cybersecurity infrastructure [17]. A systematic literature review (SLR) revealed that, between 2018 and 2019, more than 24% of the data breaches in all industries happened within the health care context [18,19].

Between 2009 and 2021, the US Department of Health and Human Services office reported 4419 health care data breaches, resulting in >314 million health care records being lost, stolen, or exposed [20]. In 2015, an estimated 113.27 million records were stolen and exposed, and in 2021 alone, the US Department of Health and Human Services also reported at least 2 health care data leaks daily [13]. The statistics clearly show an upward trend in health care data breaches over the past 10 years [21]. When considering this trend on a global scale, the number of health information breaches could potentially reach into the billions of health records. Organizations such as Vaastimo Oy Finland; National Health Service trusts in the United Kingdom; Anthem, Inc; Premera Blue Cross; and Excellus Health Plan have been victims of these threats and breaches of health information. Breaches and vulnerabilities in health care delivery, human safety, and protection of sensitive information are deeply disconcerting. However, it can be argued that research solutions are fragmented and sparse. There is a gap in the knowledge areas of health care cybersecurity in the literature and in practice regarding the vulnerability of health care systems and the reasons for cyberattacks. The argument and motivation are that a holistic approach to security is needed because humans are the weakest link in the cyberattack chain [11,22].

Coventry and Branley [6] have highlighted the need for resilience and changes in their studies on human behavior, technology, and processes as part of a holistic solution to the problem of health care system vulnerability. The information, technology, processes, objectivity and values, skills and knowledge, management systems and structure, and other resources dimensions by Heeks [23] also point out that avoiding security design reality gaps requires approaching the security functionality of a health information system as a sociotechnical system and not as a technical system. Security by design, or secure design, is an approach to cybersecurity that enables organizations to automate their data security controls and formalize the design of their infrastructure so that they can build security into their IT management processes [24,25].

In this study, a sociotechnical approach is defined as the interaction between humans and technology with the aim of creating technically efficient organizational information systems and user satisfaction [26]. Furthermore, conceptualizations of this approach are concerned with 3 primary dimensions: the social environment, technical environment, and organizational environment [27]. Sociotechnical design is identified as an approach to connect the integration of systems while ensuring that the multifaceted challenges and complexities in smart health care are well managed [28,29]. Smart health care can be defined as care that is equipped with smart IT, such as Internet of Medical Things (IoMT) devices that have the capabilities to anticipate and diagnose patient diseases; respond to treatments; guide, manage, and improve user comfort; and provide security and entertainment via hospital management systems. According to Coiera [30], “if healthcare is to evolve at a pace that will meet the needs of society, it will need to embrace the science of sociotechnical design.” Therefore, the application of a sociotechnical perspective in health care cybersecurity in this study aimed at better understanding and mitigating the multifaceted challenges and poor uptake and performance of health care system security within health care organizations.

This existing gap in knowledge and practice was a major motivation for this SLR. It is necessary to connect the fragmented research and manage this knowledge gap regarding why health care systems are vulnerable to cyberattacks as the study by Coventry and Branley [6] did not address this aspect in detail. An SLR was conducted to develop proactive cybersecurity strategies to mitigate threats and vulnerabilities that result in health care data breaches by proposing sociotechnical solutions and recommendations. Furthermore, to link human behavior, technology, and processes as highlighted by Coventry and Branley [6] and supported by the narrative review by Mohan et al [31] for further research, these 3 core areas can be interpreted as a sociotechnical framework [27]. It is essential to mitigate the increase in breaches of health information and protect health care from cybercrime and cyberattacks on critical health care infrastructure. However, none of these studies have examined why health care systems are vulnerable to attack through a sociotechnical lens. On the basis of this knowledge gap identified in the literature, the following research questions (RQs) were raised: (1) Why are health care systems vulnerable to cyberattacks? (RQ 1) (2) How can health care systems be protected? (RQ 2).

The objective of this review was to explore from a sociotechnical approach why digital health care systems are vulnerable to cyberattacks, provide sociotechnical solutions, and identify the areas of health care systems that need further improvement.

### Previous Literature Review

Regarding the existing literature on health care cybersecurity, our previous SLR identified the following review themes: (1) cybersecurity threats and trends: studies that provide solutions and insights into threats and trends have been conducted to address cybersecurity threats and trends in health care systems [2,6,11,17,32,33]; (2) cybersecurity vulnerability: some studies have also investigated the cybersecurity vulnerability of health care systems to provide solutions and future directions for health care services [22,34-36]; and (3) cybersecurity interceptions in health care: studies have also investigated cybersecurity interceptions with health care systems to protect the security posture of these systems [12,19,37]—Coventry and Branley [6] have highlighted the need for further studies on human behavior, technology, and processes to further investigate why health care systems are vulnerable and provide a holistic solution to this problem.

Therefore, there is a need for further studies to identify the reasons behind the increase in health information breaches in health care systems. This area of study through a sociotechnical lens is lacking. Accordingly, our SLR critically investigated why health care systems are vulnerable to cyberattacks and expanded this area of study from a sociotechnical point of view. This review is significant given the lack of SLRs on the areas linking human behavior, technology, and processes using a holistic approach from a sociotechnical viewpoint in this context and as the studies by Coventry and Branley [6] and Mohan et al [31] were based on narrative reviews.

## Methods

### Protocol and Registration

The PRISMA (Preferred Reporting Items for Systematic Reviews and Meta-Analyses) guidelines were followed to conduct our SLR using the checklist guide [38] (Multimedia Appendix 1). The aim of this review was to identify the reasons why health care systems are vulnerable to cyberattacks and provide sociotechnical solutions. In the planning stage of this review, a protocol for the sources of information, search strategies, study selection, criteria for eligibility, and data collection processes was created, and this review was not registered.

### Eligibility Criteria

A paper was selected for inclusion if it was published in English and comprised a full-text version of the manuscript, review paper, conference proceeding paper, report, news article or website, or white paper published between 2012 and 2022. The introduction, abstract, results, and discussion sections of the paper were checked by the authors for conformity with the study objectives and critical appraisal using the checklist guidelines before inclusion. Research papers were excluded if they were not relevant to the research areas—cybersecurity, cybercrime, ransomware, and health care. These criteria are presented in Textbox 1.

**Article inclusion and exclusion criteria.**

**Inclusion criteria**

- Study types: published peer-reviewed and original research papers (empirical and conceptual papers)
- Bibliometric study types: white papers and cybersecurity news reports in line with health care and cybersecurity
- Period: papers published between 2012 and 2022
- Language: English
- Subjects and domain: computer sciences, health care, and cybersecurity
- Requirements for paper inclusion: full-text papers.

**Exclusion criteria**

- Study types: unpublished work, editorial letters, textbooks, and research in progress
- Language: any other languages
- Subjects: studies outside the domain of cybersecurity and health care

### Information Sources

To identify original research papers and review papers on cybersecurity in health care systems published between 2012 and 2022, a total of 6 databases (Web of Science, ScienceDirect, Scopus, PubMed, Springer, and the Institute of Electrical and Electronics Engineers) and a journal (*Management Information Systems Quarterly*) were searched. Furthermore, bibliometric records such as website reports, white paper reports, and magazine reports that supported cybersecurity in health care were also collected for the review. As a means of verifying the papers identified in our search, we searched Google Scholar using a search string.

### Search Strategy

The following search string and keywords were used: (“cybersecurity” OR “cybercrime OR ransomware”) AND (“health care”) OR (“cybersecurity in healthcare”). Multimedia Appendix 2 provides more information.

### Data Extraction

A total of 70 papers were extracted and recorded in a Microsoft Excel (Microsoft Corp) spreadsheet. The extracted data included information such as author or authors, year of publication, method, problem, and solution. The first author independently charted the data and updated the table to ensure the quality of the key findings drawn from the papers based on the recommendations of the second author. Critical appraisal was conducted to ensure the quality of evidence and the relevance of the articles. The data retrieved from the selected articles were analyzed.

### Data Synthesis

The data from the literature were analyzed and synthesized using qualitative themes, which are presented in the following sections. The data were analyzed to identify the causes of vulnerabilities; solutions provided in the literature; and areas of classification based on sociotechnical, technical, and social perspectives in health care systems.

## Results

### Selection of Sources of Evidence

**Figure 1:**
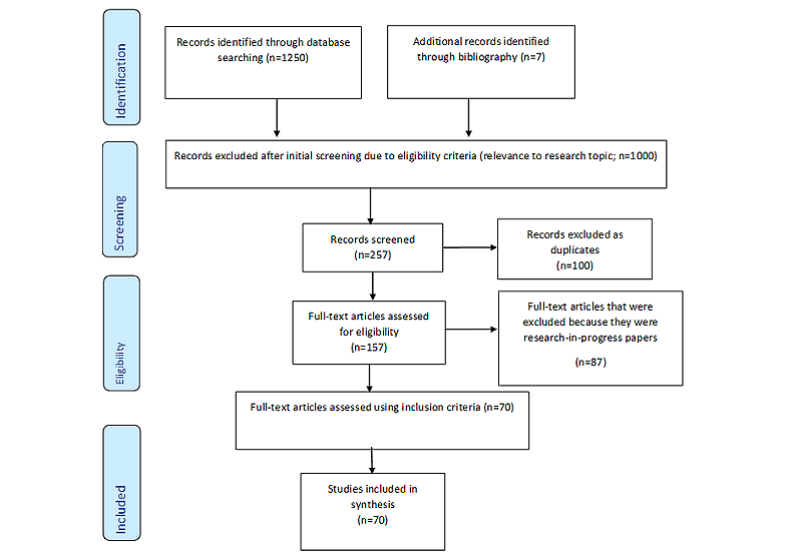
PRISMA (Preferred Reporting Items for Systematic Reviews and Meta-Analyses) flow diagram for paper selection.

A total of 1257 papers were retrieved for the screening exercises. To determine whether the papers met our inclusion criteria regarding the topic domain, we began by scanning the abstracts and titles. The papers were reviewed by reading the full texts and determining their eligibility. Duplicated papers as well as those nonrelevant to cybersecurity, cybercrime, ransomware, and health care research were excluded. Furthermore, some papers were excluded after reading them in full and discovering that they were papers on research in progress. Finally, 70 papers were included in the analysis based on the eligibility criteria. Figure 1 illustrates the selection process.

**Figure 2:**
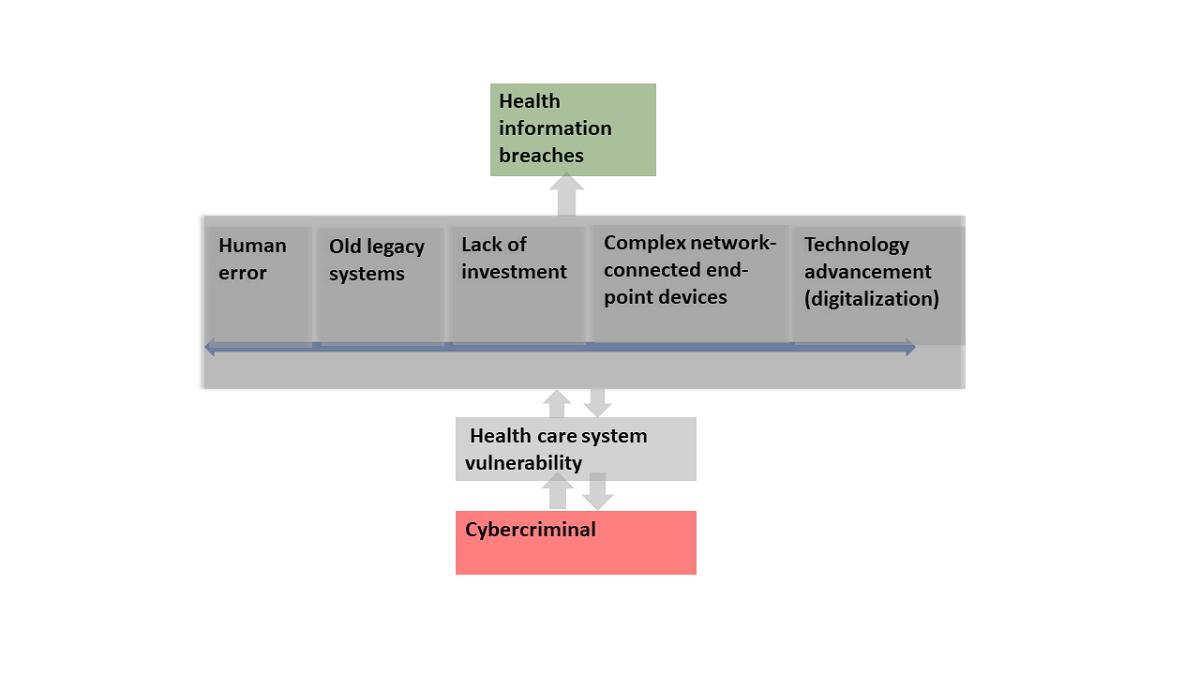
Results and insight into health care system vulnerability.

The results of the SLR show the reasons why health care systems are vulnerable to cyberattacks and health care breaches. These reasons are the 5 vulnerability themes (Figure 2 and Table 1). Furthermore, the 5 vulnerability themes were classified into social, technical, and sociotechnical approaches.

**Table 1:** Findings on health care system vulnerability categorized by themes and authors (N=70).

Vulnerabilities in health care	Type of approach	Studies, n (%)	References
Human error	Social	8 (11)	Arndt [39] Twitter [40] Mukherjee [41] Ponemon Institute [42] IBM Security [43] Scott and Wingfield [44] Jalali et al [19] He et al [36] Gordon et al [45]
Old legacy systems	Sociotechnical	11 (16)	Bouveret [46] ECRI^a^ Institute [47] Sweeney [16] Faruki et al [48] Filkins [49] Fu and Blum [50] Offner et al [2] McHugh [51] Newman [52] Scott and Wingfield [44] Tully et al [53]
Lack of investment	Sociotechnical	15 (21)	Argaw et al [11] Emsisoft Malware Lab [54,55] Branley-Bell et al [56] Information Commissioner’s Office, National Cyber Security Centre, and James M [57] Kaspersky Inc [58] PCEB^b^ [59] Rahman et al [60] Gkioulos and Chowdhury [61] Tully et al [53] Williams and Woodward [34] Coventry et al [62] Jalali et al [19,33] He et al [36] Jalali and Kaiser [37]
Complex network-connected end-point devices	Technical	36 (51)	Burns et al [63] Bouveret [46] Chua [64] Coventry et al [62] Dameff et al [8] Dienna et al [65] ECRI Institute [47] Filkins [49] Francis [66] Frost [3] Twitter [40] Giansanti [5] Handa et al [67] Offner et al [2] Klonoff [9] Lechner [68] Lewis [69] Lyon [70] McHugh [51] Mohan [71] Newman [52] Baranchuk et al [72] Perakslis [73] Peterson [74] Sajedi and Rahbar Yaghobi [75] Omotosho et al [76] Singh et al [77] Sittig and Singh [78] Snell [79] Tully et al [53] Walker [7] Williams and Woodward [34] Jalali and Kaiser [37] Jalali et al [19,33] He et al [36]
Technology advancement (digitalization)	Technical	10 (14)	Bhuyan et al [80] Coventry and Branley [6] Karambelas [4] Kruse et al [17] Raina MacIntyre et al [81] Filkins et al [82] PECB Insights [59] Jalali et al [19,33] Rodrigues et al [83]

^a^ECRI: Emergency Care Research Institute.

^b^PECB: Professional Evaluation and Certification Board.

The results also revealed that >24% of the data breaches from all industry clusters originated in the health care sector alone (Table 1) [19,21,84]. Other studies highlighted that organizations tend to spend more money on procuring new technology while committing only ≤5% of their budgets to the security of their critical health care systems [17,35]. Cybercriminals exploit health care systems due to the lack of investment, technology advancement as a result of digitalization, human error due to a lack of awareness and training, and old legacy systems, which enable cybercriminals to access valuable health information and sell it on the dark web for money and other gains [12]. The results reported a significant increase in data breaches and cyberattacks, with complex systems, IoMT devices, technology advancement, and network-connected end-point devices in complex connected heterogeneous health care systems identified as the major contributing factors.

The studies also identified a shortage of cybersecurity skills to contain cyberattacks or threats to health care organizations and systems [16]. The studies revealed that approximately 60% to 70% of health care organizations have witnessed breaches of health information without disclosure [85].

### Human Error

Human error is a significant factor in the event of a cyberattack [11,22]. This shortcoming is one of the most crucial issues in health care systems as most cybercriminals use methods such as phishing to execute attacks with just a deceitful email. This is a social problem that can be addressed from a social approach. For example, human error posed a risk to the Geneva University Hospitals [86]. Table 1 shows that 11% (8/70) of the studies acknowledged human error as the primary social reason for health care system vulnerability. Human error is attributed to a lack of skills and is a major trend in this ever-changing technological landscape, playing a role in several cybersecurity breaches [56]. From a technological point of view, a lack of expertise from humans and threats from human-related events are responsible for >70% of data fraud and breaches in business organizations (McCue, A, unpublished data, May 2008) [80] because of the value of health information on the dark web [6] and breaches in business organizations (McCue, A, unpublished data, May 2008) [80]. Furthermore, human-related threats have recently emerged as a growing concern.

### Old Legacy Systems

Old legacy systems have been the basis of system development from the dawn of the medical device, operating system, and embedded mobile device era. Legacy operating systems such as Windows ME, Windows 2000, MS-DOS, UNIX, and firmware provide the foundation for system development. However, these systems pose a significant threat to health care sectors and organizations in our current era. Table 1 shows that 16% (11/70) of the studies acknowledged the vulnerability of health care systems to attacks due to old legacy systems. Such attacks occur from a sociotechnical approach, with cybercriminals exploiting humans and technology. Many data breaches, system incompatibilities, and security risks in health care systems and sectors are associated with legacy systems. Similarly, our SLR found that 85% of medical organizations use outdated operating systems or infrastructure [12,16]. Furthermore, Fu and Blum [50] raised concerns about organizations relying on unsupported software, alluding to medical devices that run on Windows XP operating systems with service packs but lack security updates. In addition, the case of the National Health Service 2017 WannaCry malware, which interrupted health care operations and shut down numerous hospitals by infecting thousands of computers, was caused by Windows XP software [87]. The authorities had been informed about the bugs but failed to act due to negligence. When a medical device is compromised, cybercriminals use it as a gateway to abuse hospitals, health care system networks, and health information or data. Perriello [88] and Meggitt [89] highlighted another issue, *Medijack*, referring to hackers hijacking medical devices to construct a back entrance into a hospital network. As a result, the use of a network of old legacy medical devices for administrative processes and care delivery increases the opportunities for an attacker or cybercriminal to easily intrude into hospital or health care organization networks and exploit and compromise the network of medical devices and health information. In this era of rapid medical technological advancement, health care systems also lack built-in security safeguards. Legacy systems do not support new technologies, and so the network of medical equipment in intensive care units, recovery rooms, operating rooms, and electronic health records (EHRs) will lack proper and secure communication and interoperability. Outdated legacy systems and unsupported operating systems are vulnerable to high-speed attacks. Furthermore, these problems are attributable to the lack of important updates to health care infrastructure. To support our point, health and human services should provide more guidance on applying the National Institute of Standards and Technology framework to the health care industry and consider appropriate incentives that would allow health care organizations to phase out old vulnerable legacy systems [16].

### Lack of Investment

Investment in the health sector will yield better outcomes and quality health care delivery. According to our analysis and results, the health care sector suffers from underinvestment, and crucial infrastructure and training for health care cybersecurity are disregarded [6], which is one of the primary causes of the increase in sensitive health information breaches. Investment can be seen in social (human) and technical (technology) aspects. As shown in the analysis in Table 1, a total of 21% (15/70) of the studies acknowledged the lack of investment and advised both directly and indirectly regarding the necessity of cybersecurity investment in the health care industry [55,56]. The analysis acknowledged and revealed that the health care sector lagged more than other sectors in terms of health information protection and breaches. Furthermore, the findings of our SLR revealed that 80% to 85% of worldwide breaches occur in the health sector [4], whereas 45% to 90% of health care organizations have witnessed one or more threats or breaches [18,57]. Investment in critical infrastructure for health care and best practices in cyber hygiene will aid in the protection of health care systems from potential vulnerabilities. Proper investment will ensure the safeguarding of personal information and render health care systems more resilient to cyberattacks.

### Complex Network-Connected End-Point Devices

Medical end-point devices have long served as a hospital’s backbone for treatment, diagnosis, and precision-based technological applications to complement health care service operations and management. To fully exploit their potential, the medical device development pattern has shifted from traditional-based medical device system development to a network of wireless, connected end-point technological devices with built-in communications and remote connectivity. Complex network-connected end-point devices have increased the cyberattack surfaces in conjunction with their complexity and technological systems as heterogeneity in nature of medical technology has evolved. Complex network devices are classified as a technical challenge from the perspective of technical security system design. The analysis in Table 1 shows that 51% (36/70) of the studies acknowledged network-connected end-point medical devices as the most significant technical reason for health care systems’ vulnerability to cyberattacks. The operational modes continue to evolve with more interconnections between new applications and devices such as cloud-based applications, third-party software, IoMT devices, and system networks in health care environments. Lechner [68] revealed that original equipment manufacturers are now creating interconnected medical devices without incorporating proper cybersecurity features into the development life cycle of medical and end-point device systems. The vulnerability of the end point requires urgent attention; otherwise, cybercriminals will continue to use the weakness of connected devices to access personal health information. According to research and cybersecurity stakeholders, wearables, implanted devices, and sensors may become the new targets of future exploits [6,8]. As shown in Table 1, complex network-connected end-point medical devices also require medical technology security by design [72,90] as a solution strategy to protect critical health care infrastructure from breaches. In the past, medical device system development has primarily focused on critical performance and safety. Furthermore, the security aspects of these medical devices are not a factor during the planning and development process. The process indicates that developing traditional or stand-alone systems of noninterconnected devices was a suitable method for designing the traditional approach. These are the current legacy systems that lack interoperability, updates, security design, or compatibility. Furthermore, connected medical devices such as sensor-controlled drug infusion pumps, cardiac pacemakers, pulse oximeters, and network-connected x-ray machine components such as picture archiving and communication systems are vulnerable to cybersecurity threats and attacks [5]. To continue solving cybersecurity issues in medical devices, developers and actors must recognize the importance of the health care environment’s complex operations. In addition, there should be incident reports, an audit trail in the device system database, and paper-based documentation of technical vulnerabilities [34]. Medical device manufacturers such as security experts or systems integrators must address this issue because, with a single cyber vulnerability, cybercriminals or hackers can exploit medical technology connected to the internet, compromising data integrity, wearable sensor readings, protected health information, patient safety, and care outcomes [2,50]. When cyberattackers manipulate systems or deposit a virus, this could cause medical device software or systems to malfunction, resulting in abnormal effects or different readings from the systems, such as implantable medical devices that take and display incorrect readings [5,8].

### Technology Advancement (Digitalization)

Technology advancement has enabled unique access and benefits to revolutionize health care systems in terms of precision. Modern medical care now relies on health care delivery organizations, including hospitals and clinics, built on a backbone of connected computer-based infrastructure. Over the past 30 years, the expansive integration of new health care technology has changed the face of medicine [53]. However, the rapid digitalization in health care delivery, where medical devices are intertwined in a digital network setting and system to ensure the precision of health care delivery with the use of IoMT and digital devices, has created gateway access for cyberattacks, risks, and vulnerabilities [37,81]. Table 1 shows that 14% (10/70) of the studies acknowledged technology advancement due to digital transformation as the reason why health care systems are vulnerable to cyberattacks. This type of attack and vulnerability usually occur from the technical areas of cyberattacks, for example, a technology error such as glitches and design errors. One example of vulnerability is St. Joseph Hospital in California, where the health information of 31,800 patients was made public through a basic internet search engine for >1 year without anyone noticing. The underlying issue was that security settings on the medical devices were not correctly configured [91]. As technology continues to evolve, IoMT will become more inseparable in health care service delivery, which will create more vulnerabilities if health care organizations continue to disregard cybersecurity threats without proactive readiness to address them in this era of Industry 4.0. These vulnerabilities pose threats to the security and privacy of human and health information.

Studies have shown the health care sector to be unequipped and lacking in investment [11,92]. For example, the use of electronic health technology, motivated by acts such as the Meaningful Use program introduced by the US government, has compelled many health care organizations to increase the use of digital technology in health care, such as EHRs and electronic data exchange, and comply with enhanced health care delivery management. Organizations began to focus on adopting new technology and spending less on security, creating part of the problem [32]. Technological advancements and a federal policy mandate ultimatum are 2 of the causes noted in this SLR that have increased health care industry exposure to cyberattacks and breaches of health information [17]. Therefore, an organization should have proper planning; be proactive instead of reactive; and ensure the protection of health technology, information, patient privacy, and security when implementing or adopting advanced technology [17,80]. One such process is to ensure that a medical technology statement of disclosure and liability is included during the procurement, integration, and adoption of a technology. Support services and maintenance during and after procurement and installation should be part of the procurement process. Furthermore, the device manufacturer should also consider security in product development planning. Digital technology should also have the capability to monitor and collate threats and patterns and log these in a risk assessment register for analysis and improvement or threat containment.

### Causes of Vulnerabilities in Health Care Systems

**Figure 3:**
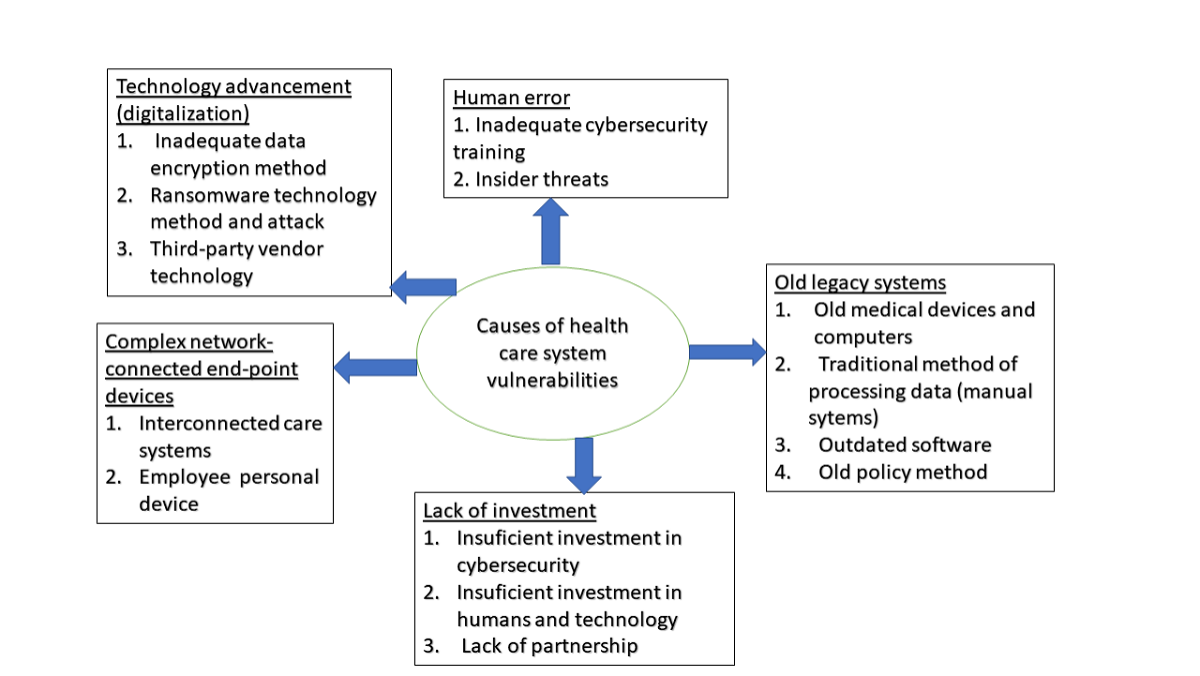
Causes of vulnerabilities in health care systems.

Figure 3 shows the causes of vulnerabilities in health care systems, which complement the findings regarding health care vulnerability, and categorizes them accordingly. The following sections address these vulnerabilities.

### How Can Health Care Systems Be Protected?

#### Overview

This study summarizes how health care systems can be protected from cyber threats and cyberattacks and presented in Table 2.

**Table 2:** Health care system protection.

Health care vulnerability and description of challenges		Proposed solutions	References	Health care cybersecurity sociotechnical areas of application	
**Human error**					Social approach
	Information breaches and identity theft	Inform human health office and owners of the data, train staff, learn to encrypt information, and have a backup plan and rollover system.	Tuttle [93]		
	Insecure behavior	Implement training.	Coventry et al [62]		
	Cyber warfare	Foster awareness and implementation of cyber hygiene. Implement data encryption, network defense solutions, and protection of premises.	Mukherjee [41]		
	Employee negligence and error	Implement training, invest in new skills for staff, and launch awareness campaign.	He et al [36]		
	Cybersecurity ethical issues, such as the disclosure and use of health information without consent	Seek patient consent and balance privacy and autonomy for health information and usability.	Loi et al [94] Christen et al [95]		
**Old legacy systems**					Sociotechnical approach
	Interoperability issues and incompatible device challenges	Procure modern devices to enable seamless synchronization of devices and networks.	—^a^		
	Interoperability issues	Implement health policy, regulation compliance, and upgrades.	—		
	Inability to update software and medical devices	Phase out legacy systems.	Sweeney [16]		
**Lack of investment**					Sociotechnical approach
	Disregard of health care cyber critical infrastructure	Invest in cyber critical systems.	Kruse et al [17]		
	Protect data, operations, and valuables	Invest in cybersecurity protection mechanisms for sensitive activities.	—		
	Design and device usability issues for processes and data security management	Invest in human behavior, technology, and organizational processes.	Coles-Kemp and Williams [96]		
**Complex network-connected end-point devices**					Technical approach
	Cyberattack on hospital health care systems	Defend the hospital with network security solutions. Have a backup and a roll-back system. Ensure that all standard policy and comprehensive guidelines are in place and always train staff to respond.	Argaw et al [11]		
	In case network-connected medical devices through the IoMT^b^ are exposed	Protect devices through assessment and extreme network defender solutions. Encrypt networks.	Frost [3]		
	Vulnerabilities due to sensor and IoT^c^ devices	Implement device simulation, security assessment, and extreme network defender solutions.	Dameff et al [8]		
	Vulnerability of end-point devices	Develop network and device security protection solutions.	Lewis [69] Singh Rayat et al [77]		
**Technology advancement (digitalization)**					Technical approach
	Lack of security in medical devices and critical infrastructure	Ensure that medical devices are designed with security before procurement and ensure that device manufacturers maintain and manage security.	Lechner [68]		
	Health care big data protection challenges	Secure life cycle model and encryption through blockchain.	Khaloufi et al [97]		
	Health care system digitalization and medical device vulnerability	Implement cyber hygiene and security in designing devices.	Coventry and Branley [6]		
	Digitalization and technology advancement vulnerability gap (digital dark alley) challenges	Update firewall installations and use a secure design approach, cloud recovery planning, and backup.	Karambelas [4]		

^a^Not applicable.

^b^IoMT: Internet of Medical Things.

^c^IoT: Internet of Things.

#### Human-Related Case Type and Challenges

The protection of health care systems from cyberattack-related vulnerabilities caused by human error, such as identity theft and health information breaches, requires by law that health care organizations inform the human health office, regulatory bodies, and data owners [93] to ensure compliance with ethical and privacy standard regulations [94,95]. A security compliance officer should also be employed to guide and ensure that proper cyber hygiene measures are in place to avoid such occurrences. It is important to ensure that health information is encrypted to assure that data are unusable and back up data offline and on the web. Furthermore, in cases in which a health care organization is saddled with challenges due to insecure human behavior, such as employee negligence, a lack of skills, and cyber warfare, the organization must ensure proper training of all staff [62] and implement awareness programs using a comprehensive guide to avert cyber threats [36,41]. This proposed solution requires a social approach in designing guidelines and training programs.

#### Old Legacy Systems Case Type and Challenges

Interoperability and compatibility challenges in medical devices stem from human-related activities within health care systems, potentially impacting the persistence of outdated legacy systems [50]. Therefore, to holistically protect health care systems, proposed solutions involve sociotechnical measures due to the old legacy in human work processes, organizational structures, and technology tasks, as mentioned by Offner et al [2]. Organizations should adhere to policies and standards linked to the old legacy, ensure proper updates and upgrades, and implement patches. Modern equipment that supports security and carries out updates must be procured to avert crises and phase out legacy systems [16].

#### Lack of Investment Case Type and Challenges

Investment in critical health care infrastructure is very important to ensure a health care ecosystem that is secure from cyberattacks and vulnerabilities. The susceptibility of health care to cyberattacks is a result of the underinvestment in and neglect of cybersecurity infrastructures. Kruse et al [17] also highlighted that a health organization invests ≤5% in cybersecurity but tends to focus on integrating and delivering care. It is important for a health care organization to invest in technology, human behavior, and processes [96] to protect sensitive and valuable health information from breaches and attacks.

#### Complex Network-Connected End-Point Devices Case Type and Challenges

The increase in health information breaches in hospitals is attributed to complex network-connected end-point devices, which are vulnerable to cyberattacks because sensor-based medical devices and system networks are interlinked and connected to the internet [8]. Internet of Things devices are vulnerable because they can be controlled through a media access control address and network. A proposed solution identified in this SLR highlighted that health care can be protected though proper encryption of data and installation of network defenders [3]. It is important that medical device simulation and assessment be performed through vulnerability analysis to ensure that devices are not tampered with or compromised [8].

#### Technology Advancement (Digitalization) Case Type and Challenges

Technology advancement has revolutionized the health care delivery process using digital technological processes. Manufactured medical devices enable patients to be diagnosed remotely, and physicians can administer care using telemedicine. However, technological advancements still lack security in the design of these devices because security is an afterthought during development, which makes them vulnerable to cyberattacks [5]. A proposed solution is that health care organizations must ensure that medical device security starts from the planning stage [68] and that device manufacturers maintain and manage security in the pre- and postmarket phases. This solution paradigm must be catalogued as a technical measure. Hospitals with modern-day smart care should leverage comprehensive guidelines and compliance with standards such as those of the International Organization for Standardization or International Electrotechnical Commission 27001 or 27002, as well as cyber hygiene to enable effective and efficient care delivery processes [4,11]. Therefore, the implementation of solutions should always adopt a sociotechnical approach [96].

### Intervention Application Areas and Domain Counts for 2012 to 2022

The selected studies from this SLR that discussed and presented knowledge interventions and solutions applied in some health care sectors between 2012 and 2022 are categorized and presented in Table 3.

**Table 3:** Intervention application areas and domain count for health care cybersecurity between 2012 and 2022 (N=70).

Vulnerability and knowledge application domain			Solution papers published in this domain between 2012 and 2022, n (%)		References
**Human error**					
	Training	12 (17)		Karambelas [4] Giansanti [5] Dameff et al [8] Argaw et al [11] Bhuyan et al [80] Offner et al [2] Holst et al [98] Branley-Bell et al [56] Chowdhury and Gkioulos [61] Khando et al [99] Coventry et al [62] Information Commissioner’s Office, National Cyber Security Centre, and James M [57]	
	Awareness	4 (6)		Walker [7] Filkins et al [82] Kaspersky Inc [58] PCEB^a^ [59]	
	Education	2 (3)		Rahman et al [60] Francis [66]	
	Intelligence information sharing	5 (7)		Bouveret [46] Winton [100] Dobuzinskis and Finkle [101] Scott and Wingfield [44] Lewis [69]	
**Old legacy systems**					
	Health policy and standards	25 (36)		Sweeney [16] Bouveret [46] Newman [52] Coles-Kemp and Williams [96] Snell [79] Emsisoft Malware Lab [54,55] Kruse et al [17] Rajamäki and Pirinen [90] The HIPAA^b^ Journal [13] Hippa [13] Khaloufi et al [97] Tuttle [93] Perakslis [73] Ponemon Institute [42,85] Tully et al [53] Bhuyan et al [80] Williams and Woodward [34] Lechner [68] McHugh [51] Burns et al [63] ECRI^c^ Institute [47] Loi et al [94] Information Commissioner’s Office, National Cyber Security Centre, and James M [57] Kaspersky Inc [58] PCEB [59]	
**Lack of investment**					
	Partnership	3 (4)		Baranchuk et al [72] Raina MacIntyre et al [81] Chua [64]	
**Complex network-connected end-point devices**					
	Participatory design science (sociotechnical)	1 (1)		Coles-Kemp and Williams [96]	
	Network security	16 (23)		Frost [3] Sittig and Singh [78] Twitter [40] Arndt [39] Bickers et al [102] Ponemon Institute [42,43] Filkins [49] Williams and Woodward [34] Zorabedian [103] Sajedi and Rahbar Yaghobi [75] Omotosho et al [76,104] ECRI Institute [47] Djenna et al [65] Mohan [71] Baranchuk et al [72] Singh et al [77]	
	Encryption	4 (6)		Mukherjee [41] Filkins [49] Mohan [71] Singh et al [77]	
**Technological advancement (digitalization)**					
	Machine learning	8 (11)		Omotosho et al [76] Zarour et al [12] Khaloufi et al [97] Reshmi [10] Faruki et al [48] Handa et al [67] Chen et al [105] Sajedi and Rahbar Yaghobi [75]	
	Blockchain	1 (1)		Bhuyan et al [80]	
	Security by design	6 (9)		Coventry and Branley [6] Lyon [70] Coles-Kemp and Williams [96] Lechner [68] Fu and Blum [50] Andrea [74]	

^a^PECB: Professional Evaluation and Certification Board.

^b^HIPAA: Health Insurance Portability and Accountability Act.

^c^ECRI: Emergency Care Research Institute.

### Knowledge Application Domains and Vulnerabilities

The vulnerabilities listed in Table 3 reveal that human error was associated with interventions linked to one of the knowledge application domains of training, awareness, education, and intelligence information sharing.

#### Training

**Figure 4:**
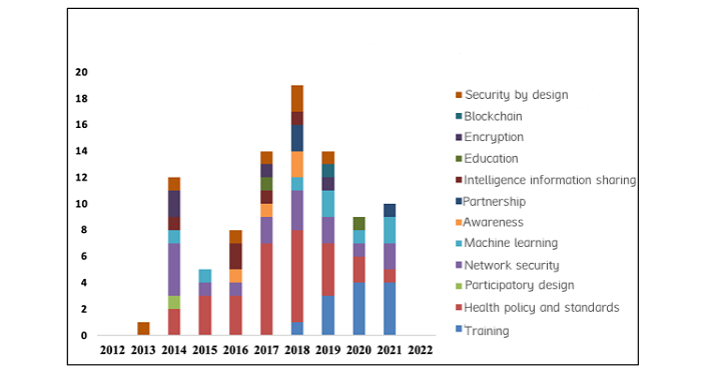
Knowledge application areas and domain count for health care cybersecurity between 2012 and 2022.

Employee training is important to avoid human factors or error challenges in health care. Table 3 shows the proposed solutions and interventions for training from 17% (12/70) of the studies. Figure 4 shows that training emerged in 2018 at 1% and increased to its peak between 2019 and 2021. However, this finding suggests the need for cybersecurity training in health care to manage human vulnerability challenges. This need is supported by the literature highlighting the importance of cybersecurity skills and education for health care professionals [16] and the need for investment in this area [17].

#### Education

The solutions presented regarding educational intervention were derived from 3% (2/70) of the studies (Table 3). Figure 4 shows that educational solutions emerged in 2017 and declined until 2020, when studies on educational intervention emerged. This finding is supported by research that shows a lack of educational skills [16]. Organizations must invest in educational training and skills to curb social and technical cybersecurity vulnerability in health care.

#### Awareness

A total of 6% (4/70) of the studies in Table 3 presented solutions on awareness to address the vulnerability of human errors. This small number of studies has shown a decline and a lack of cybersecurity awareness program in health care systems. Figure 4 similarly shows that cybersecurity awareness emerged in 2016 and reached its peak at 2 studies. This has been validated by previous studies that indicate a lack of awareness programs and training [45,62].

#### Intelligence Information Sharing

Table 3 also shows that intelligence information sharing was a solution investigated in 7% (5/70) of the studies. It can be seen that information sharing emerged in 2014 and declined in 2015 before re-emerging in 2017 and 2018 at the rate of 1 study each year. This finding also shows that health care organizations should collaborate in training and intelligence information sharing to address cybersecurity challenges in health care.

The vulnerabilities listed in Table 3 reveal that old legacy systems were associated with interventions linked to the knowledge application domain of health policy and standards.

#### Health Policy and Standards

The knowledge intervention analysis indicates that 36% (25/70) of the studies acknowledged and were linked to health policy and standards (Table 3). The analysis shows that governments and health care organizations have proposed more interventions or solutions regarding health policy and standards to regulate health care organizations. The policy studies shown in Figure 4 emerged in 2014 and continued to increase to their peak in 2018. Policies such as the Health Insurance Portability and Accountability Act, the GDPR, and the Health Information Technology for Economic and Clinical Health Act to engineer has helped to mitigate data breaches and vulnerabilities in health care organizations in addressing old legacy systems to avoid sanctions and fines in case of breaches. However, full implementation or enforcement of day-to-day monitoring in hospitals or health care organizations remains challenging.

The vulnerabilities listed in Table 1 reveal that a lack of investment was associated with interventions linked to the knowledge application domain of partnership.

#### Partnership

Partnership is key to sustaining and protecting health care systems from cybersecurity vulnerability [72]. When organizations fail to invest in critical cyber infrastructure, skills, and partnerships with governments and expert security organizations, it is likely that they will be vulnerable to cyberattacks and breaches of health information and lack the capability to protect health care systems from the vulnerability of underinvestment. Table 3 shows that partnership solutions were provided in 4% (3/70) of the studies, whereas Figure 4 shows that partnership emerged in 2018 and declined in 2021. There is a need for health care organizations to partner for better capability and structure to protect health care systems [64].

The vulnerabilities listed in Table 1 reveal that complex network-connected end-point devices were associated with interventions linked to the knowledge application domains of participatory design, network security, and encryption.

#### Participatory Design

Health care organizations and medical device manufacturers must jointly participate in designing processes and systems to avoid a sociotechnical design gap. This collaboration will help protect health care systems and increase the acceptability of organizational systems and productivity. Table 3 shows only 1 pertinent study in 2014. This infer that participatory design is one of the reasons for the vulnerabilities in complex network-connected end-point devices in health care systems. Health care systems comprise a complex environment that requires a sociotechnical and collaborative approach to addressing challenges [2].

#### Network Security

Network security solutions were covered in 23% (16/70) of the studies (Table 3). A number of intervention solution studies were conducted in this domain. As shown in Figure 4, the first increase was observed in 2014 with 4 studies, a decline to 2 studies was observed in 2017, and then the number of studies increased to 3 before a final decline to 2 studies in 2021. These studies still attest to the vulnerability of complex network-connected end-point devices, which require increased interventions to solve health care vulnerability challenges.

#### Encryption

The encryption technological solution in this review was mentioned in 6% (4/70) of the studies. There was a limited number of solutions regarding encryption intervention in this review (Figure 4). Encryption only emerged in 2014 with 2 studies, and there was a gap in studies until 2017 and 2018. This finding shows that health care organizations need to implement encryption technology to protect valuable health information from breaches and attacks [77].

The vulnerabilities listed in Table 1 reveal that technology advancement (digitalization) was associated with interventions linked to the knowledge application domains of machine learning, blockchain, and security design.

#### Machine Learning

Machine learning is a new area in which cybersecurity in health care systems is evolving. However, solutions were provided in only 11% (8/70) of the studies (Table 3). This technology surfaced in 2014 according to Figure 4. There was only 1 study in 2014 and 2015. No solutions were provided until 2018, and the number of interventions categorized under technology advancement increased from 2019 to 2021.

#### Blockchain

Blockchain technology is new and still lacking solutions according to this SLR, where only 1% (1/70) of the studies showed an effective intervention. Blockchain surfaced in 2019, as shown in Figure 4. Additional solutions and interventions are needed as this area is promising and can be categorized under technology advancement (digitalization) as the key to protecting smart health care systems.

#### Security by Design

Security by design is a strategy that demands that health care organizations implement auto-based technology to protect digital health care systems. Table 3 shows that 9% (6/70) of the studies acknowledged security by design as a solution for technology advancement to prevent vulnerability in digital systems. Figure 4 shows studies on secure design in 2013 to 2014. There were no studies in 2015, whereas in 2016 to 2019, some studies provided interventions. There is a need for more solutions in this area to protect technological advancement or digital health care systems from vulnerability [68].

### Summary of the Knowledge Application Domains and Vulnerabilities

In summary, the findings of this SLR indicate that interventions provided for the containment of health care cybersecurity vulnerabilities were limited over the past 11 years. This SLR also revealed that interventions regarding the rate of technological advancements in addressing health care cybersecurity challenges were inconsistent and lagging between 2012 and 2022. Findings also indicates that interventions in some of the mapped variables were scarce between 2012 and 2022 (Table 3). Few or no solutions are provided to address the challenges in many domains regarding health care vulnerabilities.

## Discussion

### Brief Summary of Findings

This SLR provided a synthesis of literature on cybersecurity in health care and identified the reasons why health care systems are vulnerable to cyberattacks. This review analyzed 70 published studies and identified 5 vulnerability themes of cybersecurity in health care systems and also proposed sociotechnical solutions for health care organizations.

The findings indicate that the extensive vulnerability of health care systems is due to internet-connected devices and software applications. Health care organizations face significant challenges, such as medical end-point device complexities and saturated wireless medical technology resulting in its difficulty in securing an interconnected technological landscape.

Importantly, many cyberattacks occur within this interconnected network without the health care organization’s awareness, contributing to health information breaches.

Our findings also underscore that the crucial role of investment in health care organizations is a key panacea for addressing cyberattacks and threats. Thus, lack of investment leverages the other vulnerabilities.

In addition, this study found that lack of adequate preparation for the potential threats or vulnerability in shifting to the digitalization of health care is also a contributing factor to most successful cyberattacks on health care organizations.

We found that human activity also played a major role in subjecting health care systems to cybercrimes. The decision of humans to develop medical devices, health software applications, management systems, and processes in an effective and secured manner is vital in safeguarding health information. However, there is a bit of disconnect in the human-centric design in health care system development, most importantly during the planning of procurement of medical technology and systems and the integration between health care organizations and stakeholders such as medical device developers, health care professionals, cybersecurity compliance officers, and system integration experts. Generally, the findings revealed that health care organizations lack adequate cybersecurity preparations during transitions to digitalization.

The findings also revealed that the health care cybersecurity knowledge application domain areas in Figure 4 depict that more intervention studies over the past 11 years were focused on health policy and standards.

In Table 4, solutions are proposed from a sociotechnical perspective to counteract cybersecurity vulnerabilities in health care organizations.

Further findings on the vulnerabilities and implications for future research are discussed in the following sections.

Table 4 is an integrated table that is presented in a stand-alone view for health care system solutions from a sociotechnical viewpoint.

To protect health care systems from attacks and vulnerabilities, as shown in Table 4, through the intervention of effective and noneffective studies, it can be seen that sociotechnical intervention studies classified invention most often and were the most effective. There are patterns and convergences between technical solutions and sociotechnical solutions in their domain of applications and solutions, such as a lack of investment, complex network-connected end-point devices, old legacy systems, and technology advancement, which lean toward interventions.

While we can consider human errors in human-computer interactions and technology usability from a human perspective, design and management can be approached through a sociotechnical perspective [96]. This approach also considers the final users of digital health care systems. Organizations would benefit from leveraging the sociotechnical solutions and guide in Table 4 in the case of cyberattacks attributed to human error by training all staff to respond using a comprehensive guide to avert cyber threats [62]. Challenges of technology, such as network-connected end-point devices and technology advancement for digitalization, should be addressed through network and security solutions and encryptions [6,67].

Hospitals with modern-day smart care should leverage their comprehensive guidelines and standard International Organization for Standardization or International Electrotechnical Commission 27001 and 27002 compliances.

Health care organizations should ensure and implement proper cyber hygiene to enable effective and efficient health care delivery processes [4,11]. They should increase their budget for critical cyber systems to address the lack of investment [17] and phase out old legacy systems by increasing investment. These actions will enable resilience and preparedness for future response plans and mitigations.

**Table 4:** Health care system solutions from a sociotechnical viewpoint.

Vulnerability, knowledge application domain, and description of challenge or case type				Sociotechnical lens		Effective		Not effective
**Human error**								
	**Training**							
		Ransomware or email phishing attack	Sociotechnical solution		Train and educate health care staff to use encrypted solutions for data and virus risk register; stay up-to-date on trends of virus attacks for health care systems [4,5,57]		Review cyberattacks against hospitals worldwide via training workshops through teleconferences with experts; incorrect training approach and method of delivery via teleconference [11]	
		Cyberattack on critical medical infrastructure and device breaches Ineptitude of employees regarding cybersecurity in managing health records	Sociotechnical solution Sociotechnical solution		Train and educate clinicians through simulations of hacked medical devices for patient care to heighten their awareness [8,61] Implement training for cybersecurity culture and proactive maturity resilience via human-computer interactions [2]		Review cyberattacks against hospitals worldwide via training workshops through teleconferences with experts; incorrect training approach and method of delivery via teleconference [11]	
		Insecure behavior of staff	Social solution		Assess behavior of health care staff regarding cybersecurity (insecure behavior) Apply AIDE^a^ behavior change techniques to ensure secure behavior [56,62]		—^b^	
		Health information attacks and identity theft	Sociotechnical solution		Provide employees with ISA^c^ content development material and enhance and analyze security behavior in public and private sectors Apply gamification Develop prototype game and behaviorism theory and mental model for private-sector training Apply real game and ANT^d^ for public-sector training [99]		—	
		Protection of health care system infrastructure	Sociotechnical solution		Implement cybersecurity planning and training using the CERT RMM^e^ [79]		—	
		Low digital literacy skills of employees	Sociotechnical solution		Implement essential and advanced digital literacy training via computers and smart devices [98]		—	
	**Awareness**							
		Inadequate cybersecurity awareness regarding the IoMT^f^ devices Lack of data protection compliance awareness	Sociotechnical solution		Apply cross-situational awareness model of IoMT devices for employees and management [7] Provide awareness training on HIPPA^g^ and GDPR^h^ guidelines [7,59]		—	
	**Education**							
		Employee cyberbullying Hacking and vulnerabilities of medical devices	Sociotechnical solution		Provide gamification education for web-based cyberbullies [60] Provide awareness and educational programs on the vulnerabilities of medical devices [66]		Report on pacemaker hack that led to a disconnection based on a study; the study was generalized with speculation [66]	
	**Intelligence information sharing**							
		Notification alert of threat to critical infrastructure protection Hospital management afraid to report data breach and cyberattack to protect their image	Social solution		Implement threat intelligence solution [58]. Recruit and contact compliance officer and information sharing center to report breach [46,59,100].		—	
**Old legacy systems**								
	**Health policy and standards**							
		How can we manage cybersecurity vulnerability risks	Sociotechnical solution		Implement cybersecurity risk framework [46].		—	
		Our devices lack updates	Sociotechnical solution		Provide updates and patches for legacy systems [57]		—	
		What is the lasting solution for legacy systems	Sociotechnical solution		Phase out legacy systems and procure devices with a security update that supports aftersales		—	
		Curtailing health care breaches	Sociotechnical solution		Implement GDPR and HITECH^i^ policy for medical devices and data [13,42,57,58,85].		—	
**Lack of Investment**								
	**Partnership**							
		We are concerned with the threat alerts for implanted cardiovascular medical devices. Lack of support to manage implantable devices such as pacemakers Managing threats with stakeholders to protect patients	Sociotechnical solution		Ensure security in design from manufacturers and partners for aftersales support to ensure updates with remote monitoring or interrogation [72] Ensure a partnership for a safer cardiovascular implantable device with the manufacturer’s electronic device and follow FDA^j^ and NIST-CSF^k^ guidelines [72] Health care organization should partner and implement HICP^l^ guidance [64]		Developed new biosecurity risk methods and surveillance tools from traditional methods; they lack validation [81]	
**Complex network-connected end-point devices**								
	**Participatory design science (sociotechnical)**							
		Information security design gap challenges for health care systems	—		Resolve information security design reality gap using the ITPOSOM^m^ framework by Heeks [96] and through collaboration [65].		—	
	**Network security**							
		Insecurity of connected medical devices in protecting health information Managing network security for IoMT devices	Sociotechnical solution		Install extreme network defenders to secure the network and manage IoMT devices [3]		Health record breaches in Australia are reportedly sold on the dark web; the study does not offer a solution [102]	
		Attack on critical health care cyber infrastructure	—		Develop a collaborative security approach and cybersecurity guidelines [65]		—	
		Managing complex health care network access control and authentication	Technical solution		Implement the attribute trust framework for aggregation of user attributes in a reputation system [71]		—	
		Protection of EHRs^n^ for patient safety challenge	Sociotechnical solution		Apply the 3-phase e-PSG^o^ framework [78]		—	
	**Encryption**							
		Protection of IoT^p^ devices from breaches and being compromised	Technical solution		Secure IoT devices through FHSS^q^ and RSSI^r^ techniques [77]		Anthem’s insurance health record breach report; investigation revealed that a foreign government was behind the attack, which is speculation without evidence-based facts [41]	
		Managing cloud security concerns Managing employee and patient devices on the health care network	Sociotechnical solution		Assure investment and compliance with regulatory standards and monitoring Implement policy on BYOD^s^ and apply all-layer multifactor protections for cloud systems [49]		—	
		Protecting sensitive health care data and exchange between the EHR and the cloud-based database	Technical solution		Encrypt data using lightweight cryptographic protocols; store on the cloud-based PHR^t^ [71]		—	
**Technology advancement (digitalization)**								
	**Machine learning**							
		Protecting health care systems from ransomware and other malware attacks Managing health care big data challenges	Technical solution		Implement antimalware solutions using the dynamic method [10] Implement a big data life cycle model using blockchain [80,97]		Adopting clusters to split the OCSVM^u^ machine learning algorithm; however, the study does not offer a preventative solution [67]	
	**Blockchain**							
		How can we secure health information and personal identifiable information to enable privacy and security	Technical solution		Implement information-hiding algorithms using blockchain technology [80,97]		—	
	**Secure design**							
		Formidable medical device protection Protecting health care ecosystems	Sociotechnical solution		Build in security from design planning and compliance [47,68,96] Implement stakeholder collaborative design using sociotechnical behavior [65,96]		Security trade-off on safer medical devices for patients with diabetes; proposed improvement plans are not yet implemented [70]	

^a^AIDE: Assess, Identify, Develop, and Evaluate.

^b^Not applicable.

^c^ISA: information security awareness.

^d^ANT: actor-network theory.

^e^CERT RMM: Computer Emergency Response Team Resilience Management Model.

^f^IoMT: Internet of Medical Things.

^g^HIPAA: Health Insurance Portability and Accountability Act.

^h^GDPR: General Data Protection Regulation.

^i^HITECH: Health Information Technology for Economic and Clinical Health.

^j^FDA: Food and Drug Administration.

^k^NIST-CSF: National Institute of Standards and Technology Cybersecurity Framework.

^l^HICP: Health Industry Cybersecurity Practices.

^m^ITPOSOM: information, technology, processes, objectivity and values, skills and knowledge, management systems and structure, and other resources.

^n^EHR: electronic health record.

^o^e-PSG: electronic health record–specific patient safety goals.

^p^IoT: Internet of Things.

^q^FHSS: frequency-hopping spread spectrum.

^r^RSSI: received signal strength indicator.

^s^BYOD: bring your own device.

^t^PHR: personal health record.

^u^OCSVM: one-class support vector machine.

### Implications for Future Research

#### Overview

Health care sectors have improved with policies and measures developed to control health information breaches and vulnerabilities. However, further research is needed in social and technical interception design, namely, the human factor. Managing complex end-point devices and investment on addressing health care vulnerability and breaches should be considered from a sociotechnical design and sustainability perspective.

#### Protecting Complex Network-Connected End-Point Devices

The protection of complex network-connected end-point devices for health care organizations involves several key measures. The network of interconnected medical end-point devices and the software systems that connect to the internet are becoming vulnerable to attacks and breaches. This is a growing issue; health care organizations tend to procure medical device technology without proper equipment planning and guidelines in place. This implies that security is overlooked and is not a major focus area. Examples include hospital beds connected to >10 medical devices, such as pulse oximeters, syringe pumps, and patient care monitors, which are connected devices and vulnerable to attacks [2,6].

To address this technical challenge, organizations can concentrate on developing advanced threat detection and mitigation techniques, such as network defenders tailored to intricate network-connected end-point devices in health care and the integration of artificial intelligence using machine learning algorithms to effectively identify and respond to emerging threats. Furthermore, the health care industry must take a sociotechnical approach [96] toward implementing standard guidelines and technical solutions via the protection of health care networks through planning and integrating network security protection and segmentation. In addition, health information exchange over the network should undergo steganography and encryption as a solution using blockchain technology. Therefore, the integration of a complex end-point medical device should use built-in security with alert response and communication in processes to monitor health care cybersecurity ecosystems for a healthy security posture.

Health care organizations should collaborate with security experts and health care professionals and implement user education and incidence response to catalog cyber vulnerability incidences for further analysis. The implication is that, if networks and end-point medical devices are not properly secured, this will lead to breaches of health information through the network, which will cause patient information to be hijacked by cybercriminals for political gains. Sponsored state actors may use this weakness to seize networks and systems of care delivery, demanding money from an organization before the latter can regain access. This approach will expose the health information of patients while they are receiving treatment and accessing health care services. This is an evolving challenge of the digital consequences of connected care. Building security through a design solution should be achieved from a sociotechnical approach as the human is the final user of systems of care.

Future research should focus on security by design before integrations of complex technology and design a simpler flow process with the disaggregation of complex network connections.

#### Increasing Investment in Cybersecurity

Investment in health care systems is critical to ensure the proper safeguarding of health care ecosystems from cyberattacks and vulnerabilities. To ensure efficient and secure health care, organizations should invest in human capital and technology to function effectively. An evaluation through research reveals that health care is lagging behind other sectors in terms of investment. This finding was confirmed by Kruse et al [17], who found that only 5% of health care investment is earmarked to protect health care, whereas a large percentage is allocated for health care delivery.

Insufficient investment in cybersecurity experts, awareness, and investment partnership plans will continue to subject health care employees to insecure behavior and result in a health care organization that is unprepared to mitigate cyber threats and other tactics used by attackers to disrupt evolving health care trends and patterns, particularly ransomware attacks.

Similarly, old legacy systems pose another security risk. Malicious actors can continue to exploit these systems to expose personal health information due to their limited capabilities and outdated organizational structure. Such vulnerability is worsened by a lack of investment in new cybersecurity infrastructure and computer devices to protect or process health information in a secure manner.

Health care organizations can engage in partnership with medical technology providers, application developers, and network solution integrators to develop strong systems and structures with seamless integration. Health care organizations should also develop and implement a framework for prioritizing cybersecurity investment based on risk assessments and threat intelligence. This approach can help identify the most critical areas of vulnerability within different departments, aiding organizations and policy makers in directing investments where they are most needed. Health care organizations should invest in humans and technology through training to ensure the development of necessary skills and investment in critical cyber infrastructure.

Awareness campaigns for patients and staff will help organizations recover from errors and breaches, whereas investment in technological security systems for health care will prepare health care organizations with the appropriate structure and system for resilience.

The findings presented in this paper are also highlighted in Table 4. Investment challenges in health care cybersecurity should focus on a sociotechnical approach that involves human behavior, technology, and organizational processes and should not be segregated as a separate technical or social problem. Future research should focus on security and investment in smart health care for attaining sustainability and resilience.

#### Managing Technological Advancement

Health care industries and organizations have improved over the years and are continuing to forge the development of new capabilities, technological advances, and processes to manage the multifaceted challenges of health care cybersecurity. Complexity in technology advancement and networks of digital systems increase the number of attack surfaces, where cybercriminals take advantage of the digital gateway access and execute malicious software programmed with code, such as malware to compromise digital technology and health care system networks. However, technological development necessitates a proactive approach to cybersecurity, particularly when considering security-by-design principles.

Future research projects must concentrate on important areas to protect networks, systems, and applications against vulnerabilities. Health care organizations should collaborate with medical device manufacturers as part of the planning phase of procurement requirements to ensure specifications needs before the development of medical devices technology for seamless integration. Implanted devices, for instance, should be built with security by design and continuously updated when necessary. A 2-factor authentication security for critical medical technology is also necessary. In addition, it is important that health care organizations quantify the risk, ensure that proper National Institute of Standards and Technology and GDPR standard guidelines are followed, and conduct threat modeling and simulation to evaluate the protectability of health care systems as a guideline in managing cybersecurity vulnerability.

Collaborative (sociotechnical) efforts among academia, industry, and policy makers are essential to drive this research agenda forward and create a safer digital landscape for the future.

The technology procurement requirement and collaboration should consider the integration of social and technical processes during digital technology development with health care delivery processes.

Health care organizations can adopt a blockchain technology solution for the protection of health information and other applications such as EHR systems from malicious use and insider threats.

Future research should examine the use of blockchain for health care big data protection and processes to manage cybersecurity vulnerability.

#### Containing Human Error in Cybersecurity

Humans are at the receiving end of the cyberattack chain. An example is the case of the WannaCry attack that affected 150,000 computers. It was attributed to human error because humans were warned of the attack on Windows server legacy systems but they ignored the warning by clicking on malicious email links [38,43]. When an organization fails to train humans, cybercriminals take advantage of human weakness to exploit health care systems. Today, medical device manufacturers are building devices without considering humans as the final users or a participatory (sociotechnical) design approach. This is one factor of the clinical process and security dimension to protect critical infrastructure. Another factor is that, if a system is developed and does not start with security and support human usability, it becomes stressful for a human user to navigate the systems, which could cause them techno-stress, with the likelihood of mistakes. The health sector should use the Assess, Identify, Develop, and Evaluate technique to identify areas of human weakness, develop a new training method through simulations, and offer gamification training on issues such as phishing email deception and ransomware attacks. The implication is that, if humans are not trained, they will lead organizations to disaster because cybercriminals will continue to exploit the weakness of humans to cause more damage to health care systems. The consequences will include legal issues, fines, and possibly bankruptcy for health care organizations. Proper training and awareness campaigns should be implemented. Future research should focus on developing futuristic health care cybersecurity curriculums and training.

### Practical Implications

Inadequate systems will cause health care systems and organizations to face increasing cyberattacks and setbacks in health information and patient safety. Moreover, a new trend reveals that, if implanted medical devices and technology are not protected, humans will be targeted by hackers seeking to make money or gain political power for ransom. However, implementation and adoption of the medical device security life cycle model [68] will protect medical devices, health information, patients, and organizations from harm and against future emerging threats. Thus, there is a need for the design of a cybersecurity sociotechnical framework toward sustaining smart health care systems.

### Comparison With Prior Work

Previous narrative literature reviews by Coventry and Branley [6] and Mohan et al [31] highlight the need for an integrated approach in health care systems to address cybersecurity vulnerabilities. They emphasize the need for a comprehensive approach that connects human behavior, technology, and processes in a holistic way as a best strategy to tackle vulnerabilities, although the authors did not classify human behavior, technology, and processes from a sociotechnical lens. This systematic review supports their view by building and extending the literature on cybersecurity case challenge descriptions in all the tables in this paper to integrate human behavior, technology, and processes as a sociotechnical approach [2,23,26-28]. For example, an SLR conducted by Offner et al [2] reported that health care system vulnerability is a complex sociotechnical problem. Furthermore, for a health care organization to build resilience against cyberattacks and threats to avoid cybersecurity design gaps and vulnerabilities in the health care system, a strategic approach that integrates people, technology, and processes must be adopted [23,27,31]. The aforementioned aligns with the approach adopted in this study.

Different schools of thought have highlighted the key importance of investment in technology and humans to protect health care systems from cyberattacks and threats [6,8,11,19,36,56]. This corroborates our findings that cybersecurity investment plays a main role in health care systems.

This study also revealed that complex network-connected end-point devices were mentioned several times by different schools of thought. Moreover, existing literature has opined that complex network-connected end-point devices were the most mentioned vulnerability [5,17,18,35,53].

Furthermore, technology advancement through a digital transformation evolution has created precision, and managed health care delivery [32,94]. However, more effort is still required in designing security features in health care technology. This study highlighted that security by design is required for medical device technology in health care systems [9,34,68].

Health care organizations must ensure that the design of technology evolves with a secure design approach from conception to avoid breaches of health information by external and internal attackers [24,32,68].

The sociotechnical solutions in Table 4 will aid health care organizations in being resilient in dealing with vulnerabilities and cybersecurity breaches in health care systems through a comprehensive and holistic approach. The sociotechnical perspective defines the meaning and constructs of technology, humans and processes [6,19,31,36,37]. This approach is promising and effective in dealing with health care system and cybersecurity vulnerabilities.

### Limitations

For this study, non–English-language articles on cybersecurity and health care were not included. Closed-access articles directly related to cybersecurity and health care were also not included. Textbooks linked to cybersecurity and health care were excluded. In addition, as cybersecurity is a broad topic, more time was needed for data analysis.

### Conclusions

This study conducted an SLR (PRISMA guidelines) to investigate the body of literature on cybersecurity in health care systems because of the exponential increase in health information breaches and vulnerability issues surrounding medical device technology and networks. This study also examined why health care systems are vulnerable to cyberattacks and threats.

In this review, sociotechnical solutions and mitigation strategies were proposed to protect patient health information, medical devices, and the critical cyber infrastructure of health care organizations from attacks and threats. We identified human error, lack of investment, complex network-connected end-point devices, old legacy systems, and technological advancement due to rapid digitalization as the causes of data breaches and the vulnerability of digital health care systems to attacks and threats. This study also revealed that research in the areas of education, awareness, training, collaborative partnerships, blockchain, and machine learning for health care cybersecurity is underrepresented. In addition, there was inconsistency in the publication of intervention studies. There is a gap in intervention studies published between 2012 and 2013, as shown in this SLR, as well as breaks in research publications between 2012 and 2022, as illustrated in Table 3 and Figure 4.

As shown in Table 1, of the 70 papers published between 2012 and 2022 and reviewed in this study, only 8 (11%) carried out research in the areas of human error–related perspectives where health care systems are vulnerable to attacks. This finding clearly shows that considerably more studies are required on human factors. We also identified from this review that network-connected end-point devices are the most vulnerable challenge that causes health information breaches. However, stakeholders have rolled out interventions in the areas of health policy, health care system support (network security), and training. The support and training target operational activities and health care delivery while investment in cybersecurity critical infrastructure is disregarded. Rapid technology advancement has resulted to an increasing risk of cyberattacks and threats because most manufactured connected medical devices were not built with security in mind. With the possible sociotechnical solutions in Table 4, we form conclusions about how to protect health care systems as a sociotechnical solution in relation to the gap in research on technology, human behavior, and processes.

Health care organizations must concede that efficient and effective cybersecurity cannot be addressed with a technological process only but must also evolve beyond technological operation to a sociotechnical process that calls for a comprehensive knowledge of the human elements.

The profound implication of our findings steps further from just the concept of security. It deems it necessary for a major change in the approach to health care security by shifting from a reactive measure of patching and mitigation toward an approach of proactiveness and integration of detailed mechanisms that depend on complex sociotechnical dynamics at play in the design and development processes across the health care systems.

Our review emphasized the importance of a mandatory collaboration and cross-disciplinary engagement among stakeholders in health care, technology policy, and academia. The inclusion of a team-based effort from stakeholders will foster an integrated solution that responds to the challenges of cybersecurity vulnerabilities in health care systems.

In addition, our findings also give prominence to the great significance of investment in health care systems, such as in cybersecurity technology, medical devices, networks, health care professionals, and cybersecurity professionals, in advancing health care organizations. Furthermore, investment is imperative in cybersecurity education and training programs that will provide health care professionals and organizations with the updated knowledge and skills to navigate the complexities of cybersecurity vulnerabilities constructively. Governments should provide additional financial incentives for health care organizations to facilitate cybersecurity sustainability in health care systems. Future research should explore the application of blockchain technology for safeguarding health care system data. Blockchain offers a secure decentralized architecture. Therefore, system developers should consider a human-centric design approach when integrating blockchain technology into health care systems.

By strengthening awareness culture, intelligence information sharing, and accountability in health care systems, health care organizations can equip their operations and workforce to become active front-runners in safeguarding patient data and health care critical infrastructure and assuring the confidentiality, availability, and integrity of health care systems. Consequently, our SLR implores for an exhaustive procedure regarding cybersecurity in health care that affirms and entwines the sociotechnical nature of the vulnerabilities and challenges. By merging a technical approach with human-centric strategies, health care organizations can protect health care systems from vulnerabilities and cyber threats and advance a culture of resilience, trust, and innovation in health care service delivery. The implications of this review present a sociotechnical solution for establishing more secure and resilient health care ecosystems. This paper provides health care organizations with a better understanding of and resilience to cyberattacks, threats, and vulnerabilities.

## Appendix

Multimedia Appendix 1 PRISMA (Preferred Reporting Items for Systematic Reviews and Meta-Analyses) checklist guide.

Multimedia Appendix 2 Search strategy.

## Abbreviations

EHR: electronic health record
GDPR: General Data Protection Regulation
IoMT: Internet of Medical Things
PRISMA: Preferred Reporting Items for Systematic Reviews and Meta-Analyses
RQ: research question
SLR: systematic literature review
