# Supplementary material for: Vulnerability to Cyberattacks and Sociotechnical Solutions for Health Care Systems: Systematic Review
Source: J Med Internet Res. 2024 May 31;26:e46904. doi: 10.2196/46904 (PMC11179043; doi:10.2196/46904)
Supplement: Multimedia Appendix 2 [file jmir_v26i1e46904_app2.docx]

**Multimedia Appendix 2: Annex: Literature Search Strategy Main Keyword:**

1. (Cybersecurity OR cybercrime OR Ransomware) AND (healthcare) OR (“Cybersecurity in healthcare”)
2. **1. (**Cybersecurity) AND (Healthcare) OR (“Cybersecurity in healthcare”)

| **Google Scholar database**  (Cybersecurity OR cybercrime OR Ransomware) AND (healthcare) |
| --- |

| **PubMed database**  (Cybersecurity OR cybercrime OR Ransomware) AND (healthcare) OR (cybersecurity in healthcare) |
| --- |

| **ScienceDirect (Elsevier) database**  (Cybersecurity) AND (healthcare) OR (cybersecurity in healthcare) |
| --- |

| **Web of Science (WOS)**  (Cybersecurity OR cybercrime OR Ransomware) AND (healthcare) OR (Cybersecurity in healthcare) |
| --- |

| **MIS Quarterly Journal**  (Cybersecurity) AND (healthcare) OR (Cybersecurity in healthcare) |
| --- |

| **IEEE Xplore - IEEE Electronic Library**  (Cybersecurity) AND (healthcare) OR (cybersecurity in healthcare) |
| --- |

| **SpringerLink**  (Cybersecurity) AND (healthcare) OR (Cybersecurity in healthcare) |
| --- |

| **Scopus database**  (Cybersecurity) AND (healthcare) OR (cybersecurity in healthcare) |
| --- |
